# Effect of Emulsifier Type on the Properties of SBR-Modified Cement-Based Materials

**DOI:** 10.3390/polym18091128

**Published:** 2026-05-03

**Authors:** Anhua Xu, Laifa Wang, Suining Zheng, Huiting Jia, Xinyan Wang, Yindong Xu, Huaxin Chen

**Affiliations:** 1 School of Water Resources and Civil Engineering, Qinghai Polytechnic University, Xining 810003, China; 2 Qinghai Provincial Key Laboratory of Tibet Plateau Highway Construction and Maintenance Technology, Xining 810003, China; 3 Qinghai Provincial Traffic Control Construction Engineering Group Co., Ltd., Xining 810000, China; 4 School of Materials Science and Engineering, Chang’an University, Xi’an 710061, China

**Keywords:** styrene butadiene rubber emulsion, cement-based materials, mechanical properties, durability, microstructure

## Abstract

Styrene butadiene rubber (SBR) is a commonly used polymer modifier that can improve the mechanical properties and durability of cement mortar. However, the effects of different emulsifier types on cement hydration behavior and structural evolution still need to be systematically studied. To clarify the differences among anionic, cationic, and nonionic SBR emulsions on mortar performance, three types of SBR emulsion were selected in this study. Setting time, chemically bound water, mechanical properties, chloride ion diffusion, freeze–thaw cycles, and microstructure were tested to evaluate the effects of different types and dosages on cement mortar. The results show that all three types of SBR emulsion prolong the setting time of cement paste and reduce the early hydration degree. The cationic SBR emulsion shows a more obvious effect. At 28 d, the compressive strength of mortar with 10% cationic SBR emulsion increases from 38.5 MPa to 41.2 MPa, and the flexural strength also increases. In terms of impermeability, the chloride ion diffusion coefficient decreases from 7.47 × 10^−12^ m^2^/s to 5.12 × 10^−12^ m^2^/s after adding 10% cationic SBR emulsion. After 100 freeze–thaw cycles, the compressive strength loss of ordinary mortar is 16%, while it decreases to 7.2% with 15% cationic SBR emulsion, and the mass loss is also reduced. Microstructural analysis shows that the modified mortar has a denser internal structure, improved interfacial continuity, and reduced crack development after freeze–thaw cycles.

## 1. Introduction

Cement concrete is one of the most widely used structural materials in modern civil engineering. It plays an irreplaceable role in transportation engineering, building construction, hydraulic engineering and municipal infrastructure [1]. With the continuous expansion of global infrastructure and increasingly complex service environments, concrete structures must not only meet load-bearing requirements but also maintain good crack resistance, durability and structural stability during long-term service [2,3]. However, conventional cement-based materials still have inherent limitations. They exhibit low flexural strength, high brittleness and limited deformation capacity. Under external loads, environmental attack or temperature variation, cracks are easily initiated. These cracks continue to propagate and accumulate during service, which eventually leads to durability deterioration and even structural failure [4,5].

The initiation and propagation of cracks in concrete not only weaken the mechanical performance of structures but also provide pathways for the ingress of harmful substances such as water, chloride ions and sulfates. This accelerates steel corrosion and material deterioration, which significantly shortens the service life of structures [6]. Therefore, improving flexural strength, toughness and crack resistance while maintaining the basic compressive performance of cement-based materials has become a key issue. Enhancing durability under complex service environments is also a long-term research focus in this field.

To address the high brittleness and poor crack resistance of conventional cement concrete, various modification approaches have been proposed, including fiber reinforcement, optimization of mineral admixtures, regulation by chemical additives and polymer modification [7,8,9,10]. Among these methods, polymer-modified cement-based materials are considered a promising approach because they can regulate the properties of the cement matrix at the microstructural level [11]. In this system, a certain amount of organic polymer is introduced into the cement mixture. The polymer interacts with hydration products during the hydration process, which modifies the pore structure, interfacial characteristics and overall mechanical response of cement-based materials [12].

Previous studies have shown that the incorporation of polymers can form a continuous or semi-continuous polymer film within the cement matrix. This film interweaves with hydration products and forms a spatial network with certain flexibility and toughness [13,14]. On one hand, the polymer film can fill and block capillary pores, which reduces porosity and improves the compactness of the material. On the other hand, the polymer phase has a relatively low elastic modulus and good deformability. It can buffer stress and bridge cracks under external loading, thereby suppressing crack propagation and enhancing flexural strength and crack resistance [14,15,16]. Based on these mechanisms, polymer-modified cement-based materials generally exhibit better flexural performance, impermeability and durability than ordinary cement-based materials.

With the development of polymer modification technology, various polymer emulsions have been introduced into cement-based materials, including styrene butadiene rubber (SBR), ethylene vinyl acetate (EVA), chloroprene rubber (CR) and polyacrylate emulsions (PAE) [17,18,19]. Among them, SBR emulsion has become one of the most widely used and studied polymers due to its wide availability of raw materials, mature production process, relatively low cost, and good film-forming and bonding properties [20]. Previous studies have shown that SBR emulsion can significantly improve the flexural strength and toughness of cement-based materials. It can also enhance impermeability and durability to a certain extent. Therefore, it has good application prospects in bridge deck paving, waterproof mortar, repair materials and functional mortars [21].

From a mechanistic perspective, the role of SBR emulsion in cement systems is relatively complex. On one hand, SBR emulsion gradually loses water during cement hydration and forms a polymer film. This film covers hydration products or fills pores, which significantly affects the pore structure of the cement matrix. On the other hand, an interpenetrating network can form between the polymer film and the hydration products, allowing the material to maintain strength while improving deformability and toughness [22]. In addition, previous studies have reported that the incorporation of SBR emulsion may influence the hydration kinetics of cement. It generally delays early hydration but has a limited effect on the final degree of hydration [23].

It is worth noting that emulsifiers are usually introduced during the preparation of SBR emulsion to ensure its stability in the aqueous phase. According to the type of emulsifier, SBR emulsions can be classified into anionic, cationic, and nonionic types. Different emulsifiers impart distinct surface charge characteristics to the emulsion particles. Meanwhile, the cement hydration system contains abundant Ca^2+^ ions and negatively charged hydration products. As a result, different types of SBR emulsions may exhibit significantly different interfacial interactions and hydration regulation behaviors in cement systems [24,25]. However, existing studies mainly focus on the effects of SBR dosage, polymer–cement ratio, or a single type of emulsion on the performance of cement-based materials. Systematic comparisons regarding the type of emulsifier, which is a key factor, remain limited. In particular, the intrinsic relationships among cement hydration, pore structure evolution, and macroscopic mechanical and durability properties have not been clearly clarified for different types of SBR emulsions. This limits a deeper understanding of the modification mechanism of polymer-modified cement-based materials and restricts the targeted selection and optimization of emulsion types in practical applications. Therefore, it is of great significance to systematically investigate the effects of different emulsifier types of SBR emulsions on the hydration characteristics, structural evolution, and performance responses of cement-based materials from a multi-scale perspective.

In this study, anionic, cationic, and nonionic SBR emulsions were selected to modify cement-based materials, and the effects of different emulsifier types on material performance were systematically investigated. Penetration tests and chemically bound water analysis were conducted to evaluate the influence of different SBR emulsions on the setting behavior and hydration degree of cement paste. The porosity, flexural strength, compressive strength, and freeze–thaw resistance of modified mortars was further tested to examine the variation in macroscopic properties under different emulsion systems. Meanwhile, microstructural observations were carried out to analyze the interaction between SBR emulsions and cement hydration products, aiming to clarify the underlying mechanism of how emulsifier type affects the performance of SBR-modified cement-based materials.

## 2. Materials and Methods

### 2.1. Materials

The cement used in this study was P·O 42.5 ordinary Portland cement produced by Huaxin Cement Plant, Wuhan, China. The fine aggregate was natural river sand with good gradation and a fineness modulus of about 2.3. It was washed and naturally dried before use. Tap water was used for mixing. Three types of SBR emulsions with different emulsifier types were used, namely anionic SBR (SBR1), cationic SBR (SBR2), and nonionic SBR (SBR3), all supplied by Shandong Xianyuan Chemical Technology Co., Ltd., Jinan, Shandong, China. All emulsions appeared as milky white liquids with a solid content of about 60%. Anionic SBR (SBR1) employ surfactants containing sulfonate groups, cationic SBR (SBR2) use quaternary ammonium salt surfactants, and nonionic SBR (SBR3) adopt polyoxyethylene-based nonionic surfactants. These different types of emulsifiers endow latex particles with distinct surface charge characteristics. The other physical properties are similar: the latex particles are spherical, with a particle size typically ranging from 100 to 300 nm, and a relatively uniform particle size distribution under stable emulsification conditions. Since the three emulsions originate from the same source, their particle size distribution and solid content (about 60%) are basically the same. The chemical composition of the cement is shown in Table 1.

### 2.2. Specimen Preparation

SBR-modified cement mortar and cement paste specimens were prepared separately for different tests. The SBR dosage was expressed as the polymer-to-cement ratio, set at 5%, 10%, and 15%.

For cement paste, the water–cement ratio was 0.35. In addition, since the SBR emulsion also contains 40% water, the corresponding deduction was made to the water usage in the subsequent experiments. Cement, mixing water, and SBR emulsion were mixed until a uniform paste was obtained and then cast into molds. The specimens were demolded after approximately 24 h and cured under standard conditions (20 ± 2 °C, relative humidity ≥ 95%). The prepared paste specimens were used for the determination of chemically bound water content and penetration tests. The mix proportions of the SBR-modified cement pastes are listed in Table 2.

The water–cement ratio of the cement mortar was 0.45, as with the cement paste, the water content in the SBR emulsion has been correspondingly deducted from the mixing water, and the mass ratio of cement to river sand was 1:2. During preparation, cement, mixing water, and SBR emulsion were first mixed in a mortar mixer, followed by the addition of river sand and continued mixing until a uniform mixture was obtained. The fresh mortar was then cast into molds. After resting at room temperature for 24 h, the specimens were demolded and cured under standard conditions. The mortar specimens were prepared with dimensions of 160 mm × 40 mm × 40 mm. In addition, some specimens were cast as cubes with dimensions of 70.7 mm × 70.7 mm × 70.7 mm for durability-related tests. The mix proportions of the SBR-modified cement mortars are listed in Table 3.

### 2.3. Setting Time Test

The setting time of the cement paste was determined using the Vicat method. A Vicat apparatus was used to measure the variation in needle penetration depth with time during the setting process. Timing started immediately after water was added and mixing was completed. Penetration tests were carried out at regular intervals, and the penetration depth at each time point was recorded. The initial and final setting times were determined when the penetration depth reached the specified values.

### 2.4. Chemical Bound Water Test

The degree of hydration of cement paste was characterized by measuring the chemically bound water content. Cement paste samples at different curing ages were collected and crushed. Representative portions were taken for testing. The samples were first dried in an oven at 105 °C until a constant mass was reached to remove free water. The dried mass was recorded. The dried samples were then calcined in a muffle furnace at 900 °C until a constant mass was obtained. The ignited mass was recorded. Considering that the mass loss of polymers is small and the influence is consistent, the chemically bound water content was calculated according to the following formula. This value was used to evaluate the hydration degree of the cement paste [26]. The chemically bound water content was calculated according to Equation (1).

(1) $$W=(\frac{M_{n0}-M_{n1}}{M_{n0}}-\frac{L}{1-L})\times 100\%$$

where W is the chemically bound water content (%), Mn_0_ is the mass of the cement paste after drying at 105 °C (g), Mn_1_ is the mass after calcination at 900 °C (g), and L is the loss on ignition of cement (%).

### 2.5. Mechanical Properties Test

The mechanical properties of mortar specimens were evaluated by flexural strength and compressive strength tests. Mortar prism specimens with dimensions of 160 mm × 40 mm × 40 mm were tested at different curing ages. The flexural strength was determined using a three-point bending test. After the flexural test, the two halves of the broken specimens were used for compressive strength testing. The loading rate for the flexural test was 0.05 kN/s, while that for the compressive test was 2.4 kN/s. At least three specimens were tested for each group, and the average value was reported.

### 2.6. Porosity Test

The porosity of mortar specimens was determined using the water saturation method. During the test, the specimens were immersed in water until full saturation was achieved. The saturated surface-dry mass was then measured after removing surface moisture. Subsequently, the specimens were dried in an oven at 100 °C to a constant mass, and the dry mass was recorded. Based on the mass difference between the saturated and dry states and the specimen volume, the volumetric porosity of the mortar was calculated [27]. The porosity can be determined using Equation (2).

(2) $$P=(\frac{M_{o}-M_{i}}{V}\times \rho_{w})\times 100\%$$

where M_0_ is the saturated mass of the specimen (g), M_i_ is the dry mass (g), V is the specimen volume (cm^3^), ρ_w_ is the density of water (g/cm^3^), and P is the porosity (%).

### 2.7. Chloride Diffusion Coefficient Test

The resistance of mortar specimens to chloride ion penetration was evaluated by measuring the chloride diffusion coefficient. The test was conducted using the rapid chloride migration (RCM) method. Mortar specimens were prepared as cylindrical samples with a size of Φ100 mm × 50 mm and were vacuum-saturated prior to testing, using a Φ100 mm × 200 mm specimen. First cut it exactly in the middle into two parts of the same size (Φ100 mm × 100 mm). Then, from each part, cut out a specimen with a height of (50 ± 2) mm, and use the first cut surface as the test surface exposed to the chloride ion solution. The specimens were then mounted in the migration cell and subjected to an external electric field. After the test, the specimens were split axially, and a 0.1 mol/L AgNO_3_ solution was sprayed onto the freshly fractured surface to reveal the chloride penetration front. The chloride migration depth was determined based on the color change boundary.

The chloride diffusion coefficient was calculated according to Equation (3) using the applied voltage, test duration, specimen thickness, and measured chloride penetration depth [28].

(3) $$D_{\mathrm{RCM}}=\frac{0.239\times (273+T)L}{(U-2)t}(X_{d}-0.0238\sqrt{\frac{(273+T)LX_{d}}{U-2}})$$

where D_RCM_ is the chloride diffusion coefficient (×10^−12^ m^2^/s), U is the applied voltage (V), T is the average temperature of the anolyte (°C), L is the specimen thickness (cm), X_d_ is the average chloride penetration depth (cm), and t is the test duration (h).

### 2.8. Freeze–Thaw Cycling Test

The freeze–thaw resistance of mortar specimens was evaluated using a rapid freeze–thaw cycling method. The specimens were cured in a standard curing room. At the curing age of 26 d, the specimens for the freeze–thaw test were taken out from the curing location in advance and then immersed in water at (20 ± 2) °C, with the water surface kept 20–30 mm above the top surface of the specimens. The immersion duration was 2 d, and the freeze–thaw test started at the curing age of 28 d. After taking out the specimens, their surface moisture was wiped off with a wet cloth, and the cubic specimens with dimensions of 70.7 mm × 70.7 mm × 70.7 mm were then placed in a freeze–thaw chamber for the freeze–thaw cycle test. The freezing temperature was set at −20 °C, and the thawing temperature was 20 ± 2 °C. One complete freezing–thawing process was defined as one cycle, with the duration of each cycle controlled within 4–6 h [29]. A total of 25, 50, 75, and 100 cycles were applied to evaluate the degradation behavior of the specimens under different freeze–thaw conditions.

#### 2.8.1. Mass Loss Measurement

After reaching the specified number of freeze–thaw cycles (25, 50, 75 and 100), the specimens were removed from the freeze–thaw chamber. Surface water was wiped off before weighing. The mass loss rate was calculated based on the difference between the initial mass and the mass after a given number of cycles. This parameter was used to evaluate surface scaling and structural deterioration during freeze–thaw exposure. The mass loss rate was calculated according to Equation (4):

(4) $$W_{F}=\frac{M_{F0}-M_{Fi}}{M_{F0}}\times 100\%$$

where W_F_ is the mass loss rate (%), M_F0_ is the initial mass of the specimen (g), and M_Fi_ is the mass after freeze–thaw cycles (g).

#### 2.8.2. Compressive Strength Loss Measurement

After completing the specified number of freeze–thaw cycles (25, 50, 75 and 100), compressive strength tests were conducted on the mortar specimens. The loading rate was maintained at 2.4 kN/s until failure, and the corresponding compressive strength was recorded. The compressive strength loss rate was calculated by comparing the compressive strength before and after freeze–thaw cycles, which was used to evaluate the degradation of mechanical properties under freeze–thaw action. The compressive strength loss rate was calculated according to Equation (5):

(5) $$W_{F}=\frac{M_{F0}-M_{Fi}}{M_{F0}}\times 100\%$$

where Fc_0_ is the compressive strength of the specimen at 28 d without freeze–thaw exposure (MPa), Fcn is the compressive strength after freeze–thaw cycles (MPa), and ∆f_c_ is the compressive strength loss rate (%).

#### 2.8.3. Microstructural Analysis After Freeze–Thaw Cycles

To further investigate the influence of freeze–thaw cycles on the internal structure of mortar, representative specimens were selected for microstructural analysis after 100 freeze–thaw cycles. After compressive failure, samples were taken from the inner region of the specimens and observed using scanning electron microscopy. The internal pore structure, microcrack propagation and the distribution of hydration products were systematically examined, with emphasis on the differences induced by various types of SBR emulsion.

## 3. Results and Discussion

### 3.1. Effect of Different SBR Emulsions on the Setting Time of Cement

**Figure 1:**
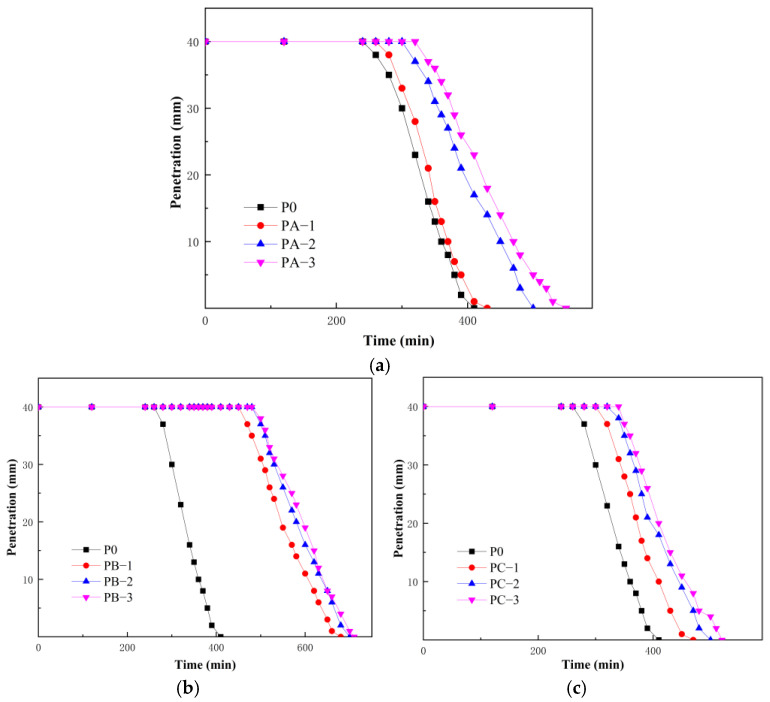
Variation in penetration depth of cement paste modified with different SBR emulsions: (**a**) anionic SBR; (**b**) cationic SBR; (**c**) nonionic SBR.

Figure 1 shows the influence of different types of SBR emulsion on the setting behavior of cement paste. The initial and final setting times of P0 were about 270 min and 400 min, respectively. After the incorporation of SBR emulsion, both the initial and final setting times of the modified cement pastes were prolonged to different extents, indicating that the polymer delayed the hydration process. For the anionic SBR emulsion, the initial setting time of PA increased from about 290 min to 340 min as the dosage increased from 5% to 15%, while the final setting time increased from about 420 min to 550 min, showing a clear dependence on dosage. The nonionic SBR emulsion also delayed the setting process, and the initial and final setting times of PC were between those of PA and PB. In contrast, the cationic SBR emulsion showed the most significant influence. The initial and final setting times of PB increased markedly even at low dosage, while the increment became less pronounced with further increase in dosage. Overall, the influence of the three emulsions on setting time followed the order: cationic > nonionic > anionic. The penetration depth of all samples decreased continuously with time and dropped more rapidly near the initial setting stage. This behavior was closely related to the formation of hydration products. As hydration proceeded, products such as C-S-H gel and Ca(OH)_2_ continuously formed, leading to a rapid increase in solid volume fraction. The cement paste gradually transformed from a fluid state to a structure-dominated viscoelastic system, resulting in a rapid increase in penetration resistance.

The difference in the retarding effect of different SBR emulsions is mainly related to the surface charge characteristics of emulsion particles and their interfacial behavior in the cement paste. Due to different emulsifiers, the emulsion particles exhibit different electrical properties in the aqueous phase. Cationic SBR emulsion particles are more likely to adsorb onto negatively charged hydration products and form a relatively dense polymer coating on the particle surface, it may be related to the formation of negatively charged C-S-H gel, which leads to a stronger blocking effect on the hydration interface. Nonionic SBR emulsions mainly influence the hydration process through steric hindrance. In contrast, anionic SBR emulsions show weaker interaction with cations such as Ca^2+^ in the highly alkaline environment, resulting in a relatively limited interfacial regulation ability [23]. Therefore, different types of SBR emulsions exhibit distinct regulation effects on hydration kinetics, which is ultimately reflected in the variation in setting time.

### 3.2. Effect of Different SBR Emulsions on the Hydration Degree of Cement

**Figure 2:**
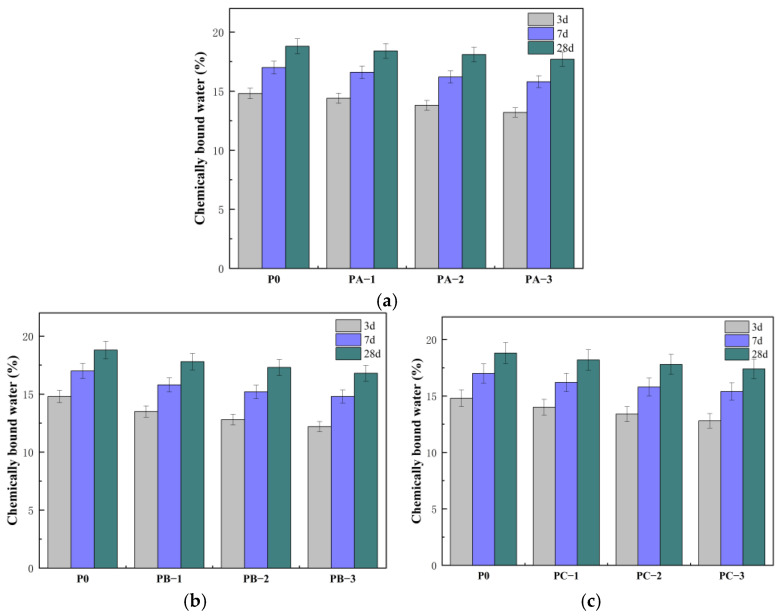
Variation in chemically bound water of cement pastes modified with different SBR emulsions: (**a**) anionic SBR; (**b**) cationic SBR; (**c**) nonionic SBR.

Figure 2 shows the variation in chemically bound water content of cement pastes modified with different SBR emulsions at different ages. Compared with P0, the chemically bound water contents of all modified cement pastes decreased, indicating that SBR emulsion retarded cement hydration. At 3 d, the chemically bound water of P0 was 14.7%, while those of PA-3, PB-3 and PC-3 were 12.8%, 11.0% and 12.2%, respectively. PB-3 showed the largest reduction, indicating that the cationic SBR emulsion exerted the strongest inhibition on early hydration. The chemically bound water of PA-3 and PC-3 was also lower than that of P0, but the reduction was less pronounced, suggesting that different emulsion types regulated hydration kinetics to different extents. This difference was mainly reflected in the early interfacial reaction stage. The cationic SBR emulsion more readily adsorbed onto negatively charged hydration products in the alkaline environment and formed a relatively continuous polymer layer, which enhanced the interfacial blocking effect and reduced the early hydration rate. During the early hydration process of cement, the high concentration of Ca^2+^ in the solution may render the surface of Ca(OH)_2_ positively charged, making it unable to adsorb cationic SBR. However, in practice, the surface charges of different hydration products in the cement hydration system vary with the hydration stage. Among them, C-S-H gel is generally negatively charged, while Ca(OH)_2_ may exhibit positive charge under specific conditions. Therefore, the adsorption behavior of cationic SBR emulsion is more likely related to the charge characteristics of negatively charged hydration products such as C-S-H or the overall interfacial zone, rather than relying solely on the surface of Ca(OH)_2_. The nonionic SBR emulsion mainly delayed ion transport through physical coating and steric hindrance. The anionic SBR emulsion was affected by charge shielding under high Ca^2+^ concentration, resulting in weaker interaction with the hydration interface and a smaller inhibition effect [23]. With increasing curing age, the chemically bound water contents of all samples increased. As the polymer–cement ratio increased, the chemically bound water at 3 d further decreased, indicating a stronger interfacial blocking effect at higher dosages. The polymer film formed on cement particles and hydration products increased the resistance to ion transport and delayed the formation of C-S-H gel and AFt. At 28 d, the chemically bound water of P0 was 18.9%, while those of PA-3, PB-3 and PC-3 were 17.9%, 17.3% and 17.7%, respectively. The difference between the modified samples and P0 became smaller compared with that at 3 d, indicating that SBR emulsion mainly affected the hydration rate rather than the final hydration degree. Hydration continued at later stages, and the early inhibition effect gradually weakened.

### 3.3. Effect of Different SBR Emulsions on the Porosity of Mortar

**Figure 3:**
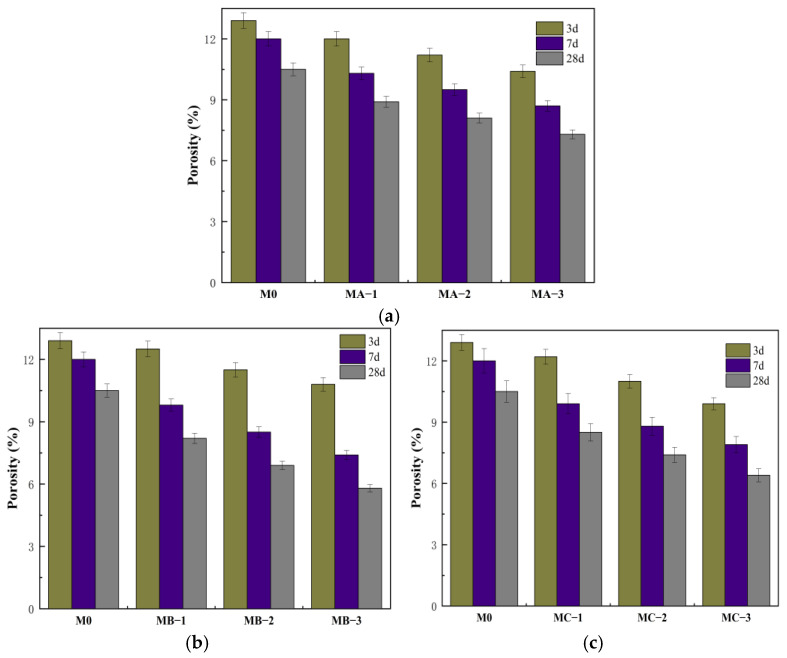
Porosity of cement mortar modified with different SBR emulsions: (**a**) anionic SBR; (**b**) cationic SBR; (**c**) nonionic SBR.

Figure 3 shows the variation in total porosity of cement mortars modified with different SBR emulsions at different ages. Compared with M0, all modified mortars exhibited lower porosity, indicating that the incorporation of SBR emulsion improved the pore structure and enhanced the compactness of the system. At 3 d, M0 showed a porosity of 12.9%, while MA-3, MB-3 and MC-3 showed 10.4%, 10.8% and 9.9%, respectively. All three emulsions reduced the early-age porosity. MC-3 showed a relatively larger reduction, while MB-3 did not exhibit a pronounced decrease. This suggests that although the cationic SBR emulsion strongly retarded hydration, its film structure was not fully developed at early age, and the pore refinement effect was still limited. At an early age (e.g., 3 d), the cationic SBR emulsion exhibits strong interfacial adsorption ability. However, this adsorption mainly acts as a coating effect on cement particles and initial hydration products, thereby significantly retarding ion migration and the hydration reaction. This process reduces the formation rate of early hydration products such as C–S–H gel, delays the formation of the structural skeleton, and keeps the matrix relatively porous at the early age. Meanwhile, the film formation of the polymer emulsion relies on gradual water loss and coalescence between latex particles. In the early stage with high water content and insufficient hydration, latex particles can hardly form a continuous and dense film structure, and their pore-filling and blocking effects are not fully exerted. Therefore, at 3 d, the hydration inhibition effect dominates in the cationic SBR system, while the structural densification effect is not yet manifested, resulting in a relatively high porosity. As the curing age increased, the porosity further decreased. At 28 d, M0 showed a porosity of 10.5%, while MA-3, MB-3 and MC-3 showed 7.3%, 5.8% and 6.4%, respectively. MB-3 showed the largest reduction, indicating that the cationic SBR emulsion provided a more significant improvement in pore structure at later age. This trend indicates a clear time-dependent effect of SBR emulsion on pore structure modification. With increasing polymer–cement ratio, the porosity further decreased, suggesting that a higher dosage promoted the formation of a more continuous polymer network. At later stages of hydration, polymer particles gradually coalesced and formed a film that filled capillary pores and the interfacial transition zone, reduced the proportion of connected pores, and refined the pore size distribution [30]. The cationic SBR emulsion, due to its stronger interfacial adsorption, more easily formed a dense coating on hydration products and therefore showed the lowest porosity at 28 d. The nonionic SBR emulsion improved the pore structure mainly through uniform film formation. The anionic SBR emulsion, affected by charge shielding, showed relatively weaker interfacial interaction and a slightly lower degree of pore refinement.

### 3.4. Effect of Different SBR Emulsions on the Mechanical Properties of Mortar

#### 3.4.1. Flexural Strength

**Figure 4:**
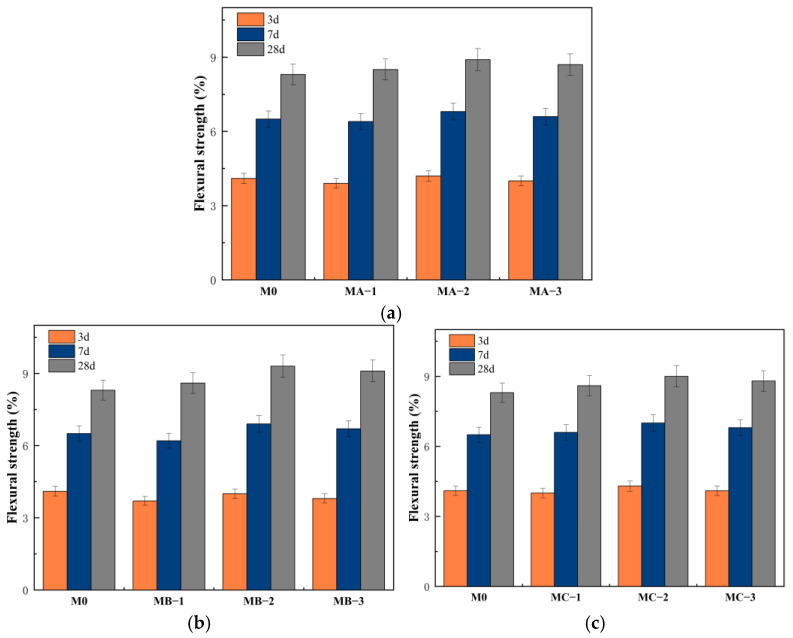
Flexural strength of cement mortar modified with different SBR emulsions: (**a**) anionic SBR; (**b**) cationic SBR; (**c**) nonionic SBR.

Figure 4 shows the variation in flexural strength of cement mortar modified with different types of SBR emulsions at different ages. The flexural strength of all samples increased with curing age. At 3 d, the flexural strength of M0 was 4.1 MPa, while MA-3, MB-3 and MC-3 were 4.0 MPa, 3.8 MPa and 4.1 MPa, respectively, which were generally close to M0, with MB-3 slightly lower. This indicated that the inhibition of hydration by the cationic SBR emulsion affected early strength development, while the anionic and nonionic emulsions had limited influence at this stage. With increasing age, the strength of all samples increased gradually. At 7 d, the flexural strength of MB-2 reached 6.9 MPa, which was higher than that of M0, indicating that the reinforcing effect of the polymer began to appear. At 28 d, the flexural strength of M0 was 8.3 MPa, while MA-2, MB-2 and MC-2 reached 8.9 MPa, 9.3 MPa and 9.0 MPa, respectively, with MB-2 showing the largest improvement. This result indicated that SBR emulsion improved the flexural performance at later stages through film formation and interfacial enhancement. The cationic SBR emulsion more easily adsorbed onto the surface of hydration products in an alkaline environment and formed a relatively continuous polymer film, which enhanced crack-bridging ability and interfacial bonding strength. The nonionic SBR emulsion improved the pore structure and interfacial transition zone through uniform film formation, leading to stable enhancement. The anionic SBR emulsion was affected by Ca^2+^ shielding, resulting in relatively weaker interfacial interaction and a smaller improvement in strength [25]. As the polymer–cement ratio increased from 5% to 10%, the flexural strength increased significantly. When it further increased to 15%, the improvement became less pronounced or slightly decreased. This indicated that an appropriate dosage facilitated the formation of a continuous polymer network, while excessive dosage might hinder hydration or cause polymer agglomeration, thereby weakening the enhancement effect. Overall, the influence of SBR emulsion on flexural strength showed a stage-dependent characteristic, with limited improvement at early ages and enhanced performance at later ages due to film bridging and pore structure refinement.

#### 3.4.2. Compressive Strength

**Figure 5:**
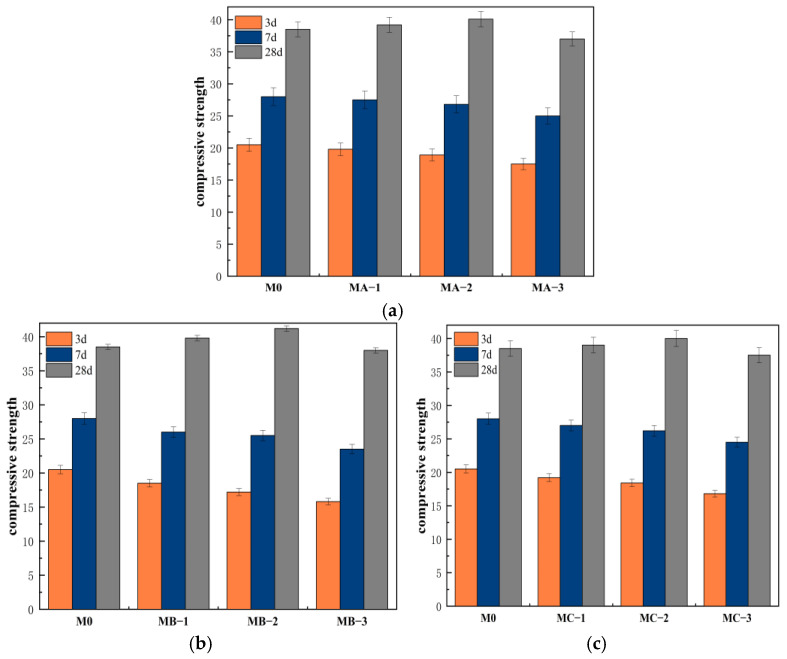
Compressive strength of cement mortar modified with different SBR emulsions: (**a**) anionic SBR; (**b**) cationic SBR; (**c**) nonionic SBR.

Figure 5 shows the variation in compressive strength of cement mortar modified with different SBR emulsions at different ages. From the figure, it can be observed that the compressive strength of all samples increased with age. Compared with M0, the early compressive strength of the mortar decreased after the incorporation of SBR emulsion, while a certain improvement was observed in later stages. At 3 d, the compressive strength of M0 was 20.5 MPa, while those of MA-3, MB-3 and MC-3 were 17.5 MPa, 15.8 MPa, and 16.8 MPa, respectively. Among them, MB-3 showed the largest reduction, indicating that the cationic SBR emulsion had a more significant effect on early hydration. As the dosage increased, the compressive strength of the three modified mortars showed a downward trend, indicating that higher dosages of emulsion enhanced the inhibitory effect on hydration and structure formation. At 7 d, the compressive strength of all samples further increased. MA-2, MB-2 and MC-2 reached 26.8 MPa, 25.5 MPa, and 26.2 MPa, respectively, with a noticeable reduction in the gap compared to M0. This suggests that as the hydration reaction continued, the inhibitory effect of the emulsion on the hydration rate weakened, and the structure gradually improved. At 28 d, the compressive strength of M0 was 38.5 MPa, while those of MA-2, MB-2, and MC-2 were 40.1 MPa, 41.2 MPa, and 40.0 MPa, respectively, with MB-2 performing the best. In contrast, MA-3, MB-3, and MC-3 showed 37.0 MPa, 38.0 MPa, and 37.5 MPa, respectively, with no further increase, indicating that excessive dosages were unfavorable for the development of compressive strength. From the perspective of type differences, the cationic SBR emulsion had a greater impact on compressive strength at early stages, but its effect on strength improvement was more pronounced at later stages. The nonionic SBR emulsion showed more stable performance, while the anionic SBR emulsion showed a relatively small overall change. In general, the influence of SBR emulsion on compressive strength exhibited clear stage-dependent characteristics. Early on, hydration inhibition slightly reduced strength, but later on, film formation and interfacial strengthening led to structural optimization, resulting in an increase in compressive strength at appropriate dosages. The differences between different emulsion types were primarily due to differences in interfacial adsorption capacity and film formation efficiency.

### 3.5. Effect of Different SBR Emulsions on Chloride Permeability of Mortar

**Figure 6:**
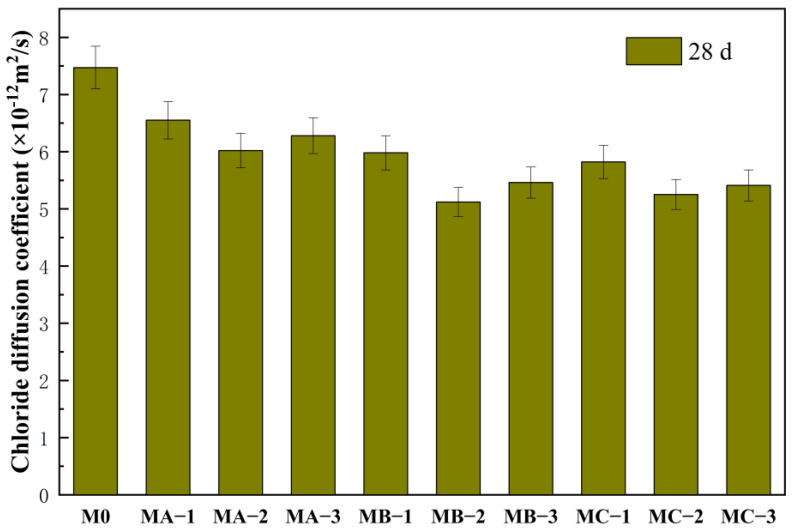
Chloride diffusion coefficient of cement mortar modified with different SBR emulsions at 28 d.

Figure 6 presents the chloride diffusion coefficients of all mortar samples at 28 d. The chloride diffusion coefficient of M0 was 7.47 × 10^−12^ m^2^/s. After the incorporation of SBR emulsions, the chloride diffusion coefficients of all modified mortars decreased significantly. The values of MA-2, MB-2, and MC-2 were 6.02 × 10^−12^ m^2^/s, 5.12 × 10^−12^ m^2^/s, and 5.25 × 10^−12^ m^2^/s, respectively, all lower than that of M0. When the dosage increased to 15%, the chloride diffusion coefficients showed a slight increase compared with those at 10%. The reduction in chloride diffusion coefficient indicates an improvement in resistance to ion penetration. In the later stage of hydration, the SBR emulsion gradually formed a polymer film structure, which was distributed within pores and interfacial regions. This structure increased the tortuosity of the transport path and enhanced impermeability. At a dosage of 10%, the polymer film was more continuous, which favored the formation of a stable barrier structure. With further increase in dosage, local aggregation of polymer reduced structural uniformity, resulting in a less pronounced improvement. Among the different types, MB-2 showed the lowest chloride diffusion coefficient, indicating that the cationic SBR emulsion was more effective in enhancing impermeability. This is related to its stronger adsorption on hydration products in an alkaline environment, which leads to the formation of a denser interfacial layer. MC-2 followed, as the nonionic SBR emulsion mainly improved impermeability through uniform film formation. MA-2 showed a relatively smaller reduction, as the anionic SBR emulsion experienced charge shielding under high Ca^2+^ conditions, resulting in weaker interfacial regulation. Overall, the improvement in impermeability varied significantly with emulsion type, with the cationic SBR emulsion showing the most pronounced effect.

### 3.6. Effect of Different SBR Emulsions on the Freeze–Thaw Resistance of Mortar

#### 3.6.1. Mass Loss

**Figure 7:**
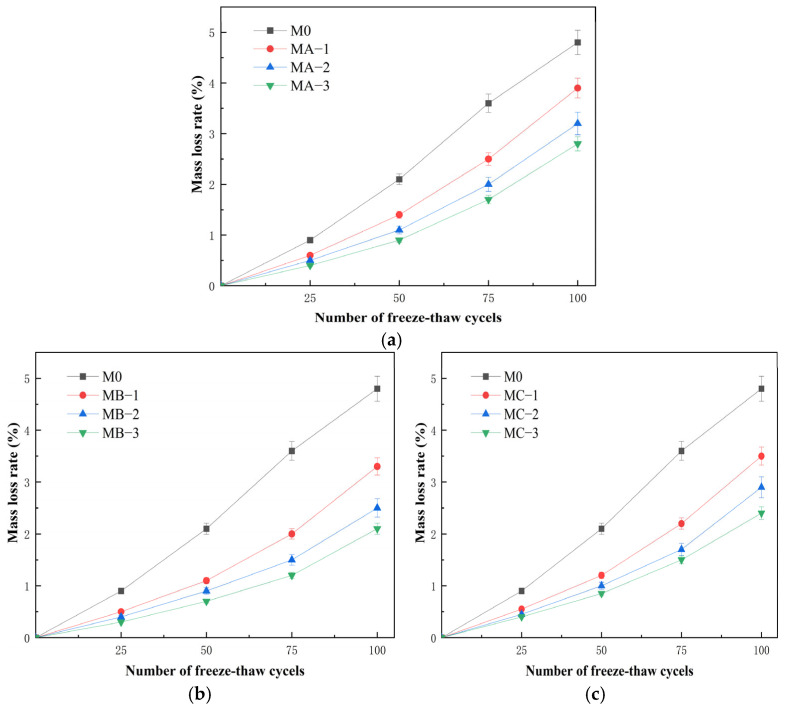
Variation in mass loss rate of cement mortar modified with different SBR emulsions during freeze–thaw cycles: (**a**) anionic SBR; (**b**) cationic SBR; (**c**) nonionic SBR.

Figure 7 shows the variation in mass loss rate of cement mortar modified with different types of SBR emulsions during freeze–thaw cycles. As the number of cycles increased, the mass loss rate of all specimens gradually increased. After 100 cycles, the mass loss rate of M0 reached 4.8%, which was significantly higher than that of the modified mortars. The mass loss rates of MA-3, MB-3 and MC-3 after 100 cycles were 2.8%, 2.1% and 2.4%, respectively. Overall, the incorporation of SBR emulsion reduced the mass loss rate, and the freeze–thaw resistance improved as the dosage increased from 5% to 15%. In terms of emulsion type, the cationic SBR emulsion showed the most stable performance. MB-2 and MB-3 maintained relatively low mass loss rates even after 75 and 100 cycles, indicating better structural stability under repeated freeze–thaw action. The nonionic SBR emulsion showed a slightly weaker effect, while the anionic SBR emulsion exhibited the smallest improvement, although it still performed better than M0. The improvement in freeze–thaw resistance is mainly attributed to the polymer film formed during later hydration. This film distributes within pores and interfacial regions and helps buffer internal stress during freeze–thaw cycles, thereby reducing damage caused by water freezing and expansion. In addition, the polymer enhances the interfacial transition zone and suppresses the propagation of microcracks, which reduces surface scaling and mass loss. Due to its stronger interfacial adsorption ability, the cationic SBR emulsion can maintain a more stable interfacial structure under freeze–thaw conditions, resulting in the lowest mass loss rate. Overall, SBR emulsion improves the freeze–thaw resistance of cement mortar, and the enhancement becomes more pronounced with increasing dosage, with the cationic type showing the best performance.

#### 3.6.2. Compressive Strength Loss Rate

**Figure 8:**
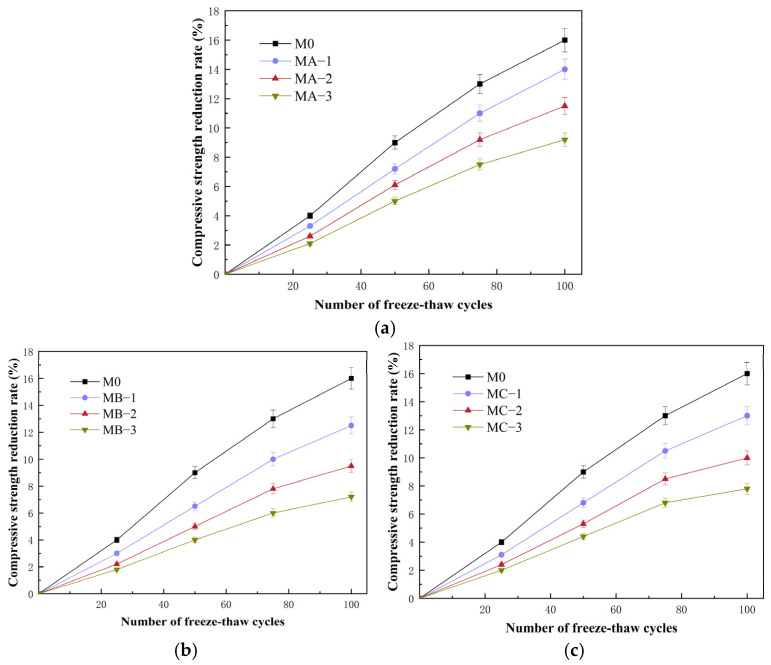
Variation in compressive strength loss rate of cement mortar modified with different SBR emulsions during freeze–thaw cycles: (**a**) anionic SBR; (**b**) cationic SBR; (**c**) nonionic SBR.

Figure 8 shows the variation in compressive strength loss rate of cement mortar modified with different SBR emulsions under freeze–thaw cycles. The compressive strength loss rate of all specimens increased with the number of cycles. After 100 cycles, the loss rate of the control mortar reached 16%. After incorporating SBR emulsion, the strength loss was significantly reduced. The values of MA-3, MB-3 and MC-3 were 9.2%, 7.2% and 7.8%, respectively. Increasing the polymer–cement ratio helped to reduce strength deterioration, and the cationic SBR emulsion exhibited the lowest loss level. Compared with mass loss, compressive strength loss better reflects the accumulation of internal damage. During freeze–thaw cycles, water in pores repeatedly freezes and melts, leading to cyclic volume changes and continuous initiation and propagation of microcracks. In the control mortar, cracks gradually coalesced within the skeleton, resulting in a clear reduction in load-bearing capacity. After adding SBR emulsion, a flexible polymer network formed within the hardened matrix. This improved deformation compatibility and provided a bridging effect at early crack stages, which slowed crack propagation and reduced strength degradation. The differences among emulsion types were mainly related to interfacial stability. The cationic SBR emulsion more readily interacted with hydration products in the alkaline environment and formed a more stable interfacial structure, which better maintained the integrity of the internal skeleton under freeze–thaw conditions. The nonionic SBR emulsion showed a moderate improvement, while the anionic type was relatively weaker, but all were superior to the control mortar. Overall, SBR emulsion effectively reduced compressive strength deterioration induced by freeze–thaw cycles, and the improvement became more pronounced with increasing dosage, with clear differences among emulsion types.

#### 3.6.3. Microstructure

**Figure 9:**
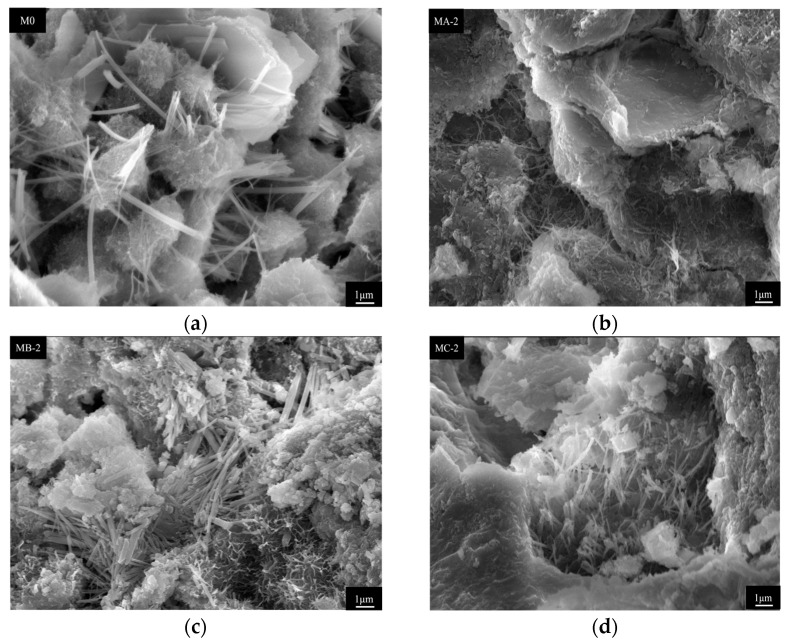
Microstructure of cement mortar modified with different SBR emulsions after 100 freeze–thaw cycles: (**a**) M0; (**b**) MA-2; (**c**) MB-2; (**d**) MC-2.

Figure 9 shows the microstructure of cement mortar modified with different SBR emulsions after 100 freeze–thaw cycles. The microstructural analysis in this study mainly serves to assist in interpreting macroscopic performance changes, and the conclusions are mainly supported by the results of mechanical properties, pore structure and durability tests. Structural deterioration was observed in all samples, while the damage features varied significantly. The control mortar exhibited a loose structure. A large number of needle-like crystals were exposed. Hydration products were poorly bonded, and microcracks and pore expansion were observed in local regions, indicating that repeated freeze–thaw cycles damaged the internal skeleton. Based on the morphological characteristics of the components, the acicular crystals are presumed to be AFt, although this identification requires further verification by means such as EDS. The acicular products, which may be ettringite (AFt) crystals, were irregularly distributed, and local debonding occurred at the interface. After incorporating the anionic SBR emulsion, the structural integrity was improved to some extent. Needle-like hydration products were still observed in MA-2, but their distribution became more uniform. Partial filling appeared along pore edges, while microcracks were still present at local interfaces, suggesting limited improvement under freeze–thaw action. The microstructure of MC-2 was denser. Hydration products were closely packed, pore walls were relatively intact, and no obvious through-cracks were observed. Continuous film-like structures were locally distributed between hydration products, indicating that the polymer remained stable during freeze–thaw cycles. MB-2 showed the most intact microstructure. Hydration products were tightly arranged, pore edges were clear and continuous, and no obvious spalling or penetrating cracks were observed. Needle-like crystals were partially embedded in the matrix, and the interface exhibited a smoother transition, indicating better structural stability under cyclic freeze–thaw action. Overall, freeze–thaw cycles caused severe damage in the control mortar, while the incorporation of SBR emulsions improved structural integrity. Differences were observed among emulsion types. The cationic SBR emulsion showed superior performance in maintaining interface stability and structural continuity. This is consistent with the results of mass loss and compressive strength loss, indicating that SBR emulsion enhances freeze–thaw resistance by improving internal stability.

## 4. Conclusions

This study systematically investigated the effects of anionic, cationic and nonionic SBR emulsions on the hydration behavior, mechanical properties and durability of cement mortar. The main conclusions are as follows:

(1) All types of SBR emulsion prolonged the setting time of cement paste and reduced the early hydration degree compared with the control cement paste. The cationic SBR emulsion showed a more pronounced effect on early hydration. At a dosage of 15%, the chemically bound water of the modified cement paste at 3 d decreased from 14.7% for the control cement paste to 11.0%. With increasing age, the bound water content of all samples gradually increased and approached that of the control cement paste.

(2) The anionic, cationic and nonionic SBR emulsions improved the mechanical properties of cement mortar at appropriate dosages compared with the control mortar. The cationic type showed a more significant enhancement. At 28 d, the compressive strength of mortar with 10% cationic SBR emulsion increased from 38.5 MPa for the control mortar to 41.2 MPa, and the flexural strength was also improved. Further increase in dosage led to a slower strength gain.

(3) All three types of SBR emulsion improved the pore structure and enhanced the impermeability of cement mortar compared with the control mortar. The cationic SBR emulsion exhibited a more pronounced reduction in chloride ion transport. At 10% dosage, the chloride ion diffusion coefficient at 28 d decreased from 7.47 × 10^−12^ m^2^/s for the control mortar to 5.12 × 10^−12^ m^2^/s, accompanied by a denser pore structure.

(4) The anionic, cationic and nonionic SBR emulsions all improved the freeze–thaw resistance of cement mortar compared with the control mortar. The cationic SBR emulsion showed the most stable performance. After 100 freeze–thaw cycles, the compressive strength loss of the control mortar was 16%, while it decreased to 7.2% for mortar with 15% cationic SBR emulsion. The surface deterioration was significantly reduced.

## 5. Prospect

This study only investigated the mechanical properties at 28 d and the performance under single-factor freeze–thaw cycles for SBR-modified cement-based materials, and thus has certain limitations in research scope. For practical engineering applications such as bridge deck paving, waterproof mortar and repair materials, the long-term service performance at 90 d, 180 d and longer ages, as well as the evolution laws under complex deterioration mechanisms including coupled freeze–thaw and chloride attack, coupled load and environmental effects, still need to be further revealed. The conclusions obtained in this paper are mainly applicable to short-term laboratory conditions and should be verified in combination with actual environments for engineering applications. Future research can systematically focus on long-term performance evolution, multi-factor coupled durability, and service characteristics under real bridge loads, so as to provide more comprehensive theoretical and data support for the engineering application and promotion of SBR-modified cement-based materials.

## Figures and Tables

**Table 1:** Chemical composition of cement (%).

	CaO	SiO_2_	Al_2_O_3_	Fe_2_O_3_	MgO	SO_3_	LOI
Cement	60.28	22.18	5.71	3.63	2.65	2.88	1.47

**Table 2:** Mix proportions of cement pastes.

	Cement	Water (%)	SBR1 (%)	SBR2 (%)	SBR3 (%)
P0	100	35	—	—	—
PA-1	100	33	5	—	—
PA-2	100	31	10	—	—
PA-3	100	29	15	—	—
PB-1	100	33	—	5	—
PB-2	100	31	—	10	—
PB-3	100	29	—	15	—
PC-1	100	33	—	—	5
PC-2	100	31	—	—	10
PC-3	100	29	—	—	15

**Table 3:** Mix proportions of cement mortars.

	Cement	Sand	Water (%)	SBR1 (%)	SBR2 (%)	SBR3 (%)
M0	100	300	45	—	—	—
MA-1	100	300	43	5	—	—
MA-2	100	300	41	10	—	—
MA-3	100	300	39	15	—	—
MB-1	100	300	43	—	5	—
MB-2	100	300	41	—	10	—
MB-3	100	300	39	—	15	—
MC-1	100	300	43	—	—	5
MC-2	100	300	41	—	—	10
MC-3	100	300	39	—	—	15

## Data Availability

The original contributions presented in this study are included in the article. Further inquiries can be directed to the corresponding authors.
